# Antiretroviral treatment interruption and resumption within 16 weeks among HIV-positive adults in Jinan, China: a retrospective cohort study

**DOI:** 10.3389/fpubh.2023.1137132

**Published:** 2023-05-09

**Authors:** Jing Ma, Yan Jin, Kedi Jiao, Yao Wang, Lijie Gao, Xinrui Li, Wei Ma

**Affiliations:** ^1^Department of Epidemiology, School of Public Health, Cheeloo College of Medicine, Shandong University, Jinan, Shandong, China; ^2^Institution for Acquired Immunodeficiency Syndrome (AIDS)/Sexually Transmitted Diseases (STD) Control and Prevention, Jinan Center for Disease Control and Prevention, Jinan, Shandong, China; ^3^Vanke School of Public Health, Tsinghua University, Beijing, China

**Keywords:** HIV, antiretroviral therapy, treatment interruption, resumption, retention

## Abstract

**Background:**

Treatment interruption has been found to increase the risk of opportunistic infections and death among HIV-positive adults, posing a challenge to fully realizing antiretroviral therapy (ART). However, it has been observed that short-term interruption (<16 weeks) was not associated with significant increases in adverse clinical events. There remains a dearth of evidence concerning the interruption and resumption of ART after short-term discontinuation in China.

**Methods:**

HIV-positive adults who initiated ART in Jinan between 2004 and 2020 were included in this study. We defined ART interruption as more than 30 consecutive days off ART and used Cox regression to identify predictors of interruption. ART resumption was defined as a return to ART care within 16 weeks following discontinuation, and logistic regression was used to identify barriers.

**Results:**

A total of 2,506 participants were eligible. Most of them were male [2,382 (95%)] and homosexual [2,109 (84%)], with a median age of 31 (IQR: 26–40) years old. Of all participants, 312 (12.5%) experienced a treatment interruption, and the incidence rate of interruption was 3.2 (95% CI: 2.8–3.6) per 100 person-years. A higher risk of discontinuation was observed among unemployed individuals [adjusted hazard ratio (aHR): 1.45, 95% CI: 1.14–1.85], with a lower education level (aHR: 1.39, 95% CI: 1.06–1.82), those with delayed ART initiation (aHR: 1.43, 95% CI: 1.10–1.85), receiving Alafenamide Fumarate Tablets at ART initiation (aHR: 5.19, 95% CI: 3.29–8.21). About half of the interrupters resumed ART within 16 weeks, and participants who delayed ART initiation, missed the last CD4 test before the interruption and received the “LPV/r+NRTIs” regimen before the interruption were more likely to discontinue treatment for the long term.

**Conclusion:**

Antiretroviral treatment interruption remains relatively prevalent among HIV-positive adults in Jinan, China, and assessing socioeconomic status at treatment initiation will help address this issue. While almost half of the interrupters returned to care within 16 weeks, further focused measures are necessary to reduce long-term interruptions and maximize the resumption of care as soon as possible to avoid adverse clinical events.

## 1. Introduction

Antiretroviral therapy (ART) has revolutionized the management and clinical outcomes among people living with HIV (PLHIV) and transformed HIV infection into a manageable chronic disease (1–4). As a life-long therapy, sustained optimal use of ART is necessary to ensure maximum therapeutic benefits and reduce onward transmission (5).

Nevertheless, treatment interruption (TI) is a common phenomenon in clinical practice, often occurring due to treatment fatigue or in an attempt to minimize side effects and ART toxicities (6, 7). A previous study has addressed TI as a possible strategy to offset treatment fatigue, enhance the quality of life, limit adverse events, and reduce costs (8). However, TI has been found to increase the risk of HIV disease progression, opportunistic infections, and death among adults in prospective clinical trials and observational studies (6, 9–12); thus, structured TI among HIV-positive adults is no longer recommended (13–15).

Despite recognition of the detrimental effects of TI, many studies continued to report on the high prevalence of TI in both developed and developing countries, which ranged from 6 to 51% (9, 16). Studies on TI in European countries have reported incidence rates around 2.5–3.5/100 person-years (17, 18). Previous studies have identified some factors associated with TI. For example, some studies showed unemployment, low income, and low education as risk factors for the interruption (16, 19). However, the association of age, gender, CD4 count and ART regimen with TI was inconsistent across studies (9, 11, 20–23). Therefore, studies are needed to elucidate further the factors associated with TI to identify PLHIV who need more targeted support for maintaining optimal adherence.

The resumption of treatment for those who discontinued therapy is another issue of interest. Previous study have shown that male, older age, and individuals with a CD4 cell count < 200 cells/μL at ART initiation were more likely to resume treatment (24). A secondary analysis of SMART participants demonstrated that short-term TI of <16 weeks was not associated with significant increases in adverse clinical events (25). Recent reports suggested that short-term TI did not lead to significant expansion of the HIV reservoir or prolonged immunologic consequences after ART re-initiation (26–29). But very few studies have examined the resumption rate and how it correlates with the resumption of treatment (within 16 weeks) after interruption.

Given the challenges of maintaining PLHIV on effective long-term ART, a better understanding of the incidence of interruption, particularly long-term discontinuation, and associated factors may allow better identification of PLHIV who need stronger support to maintain adherence to ART. Thus, we conducted a retrospective cohort study to explore the incidence rate of the first TI among adult PLHIV and identify TI determinants. Considering that short-term TI is not associated with adverse clinical events (25), we assessed rates and barriers of ART resumption within 16 weeks among interrupters.

## 2. Methods

### 2.1. Study design and population

This retrospective observational cohort study looked at HIV-positive people who started ART through China's National Free Antiretroviral Treatment Program (NFATP) in Jinan, China, between 2004 and 2020. The inclusion criteria were: (1) age of 18 years or older, (2) initiation of ART at Jinan for the first time between 2004 and 2020, (3) having attended at least one follow-up after initiating ART, and (4) did not transfer out to other cities. Subjects were followed from the date of ART initiation until the time of (1) first treatment interruption, (2) June 30, 2021 (minimum 6 months follow-up; maximum 5 years follow-up). Participants who had TI were then followed from the date of treatment interruption until the time of (1) ART resumption and (2) December 31, 2021 (minimum 6 months; maximum 12 months).

### 2.2. Data source and management

The data used in this study were collected from the NFATP. When each patient confirmed a diagnosis of HIV and consented to begin ART, a standard assessment was performed, during which baseline information, such as sociodemographic status (age, gender, marriage status, education level, employment) and HIV/ART-related characteristics (e.g., HIV transmission route, WHO stage, CD4 cell count, treatment facility, and ART regimen) were collected. Follow-up visits were conducted at 0.5, 1, 2, and 3 months after ART initiation and every 3 months after that. The attendance date and ART regimen were recorded at each follow-up visit. Patients in ART care were provided with free CD4 testing once a year. Detailed information on the Chinese national free antiretroviral treatment cohort has been published elsewhere (30).

### 2.3. Outcomes and definitions

The first outcome, the first treatment interruption, was defined as an ART interruption for at least 30 consecutive days since ART initiation, in line with other studies (19, 31). Duration of discontinuation of fewer than 30 days was excluded as short interruptions are common, sometimes unavoidable (for example, after side effects) (32). TI was identified through prescription information extracted from clinical records in NFATP. Here we talked about treatment interruptions, not follow-up interruptions. The date for treatment interruption was defined as the last pick-up date plus days of supply of picked drugs. If discontinuation didn't occur, data were censored on June 30, 2021. Care records were analyzed up to July 30, 2021, to ensure that there was a 30-day period of hindsight required to define a TI.

The second outcome was getting back on ART after a short break, defined as getting back on ART within 16 weeks of the first break. Returning to HIV care was defined as a prescription record in NFATP. The analysis period began from the date of the first TI and was censored at the date of returning to ART care or December 31, 2021, whichever occurred first.

### 2.4. Covariates

Sociodemographic factors included gender (male, female), age at ART initiation (as a continuous variable and further categorized into three groups: 18–30 years, 31–40 years, and 41 or above), educational level (middle school and below, high school and above), marital status (unmarried, married/ever married), and employment status (employed, unemployed). The HIV-related characteristics included HIV transmission route (heterosexual, homosexual or other) and WHO staging (1, 2 and above). ART-related characteristics included interval time from HIV diagnosis to ART initiation (≤30, 31–90, >90 days), treatment facility [hospital settings, Center for Disease Control and Prevention (CDC)], CD4 cell counts, and ART regimen at baseline [tenofovir (TDF) + nucleoside reverse transcriptase inhibitors (NRTIs) + non-nucleoside reverse transcriptase inhibitors (NNRTIs), zidovudine (AZT) + NRTIs + NNRTIs, Elvitegravir/cobicistat/Emtricitabine/Tenofovir alafenamide fumarate (E/C/F/TAF) and others].

For analysis of ART resumption, in addition to the above sociodemographic information and HIV-related characteristics, the year of interruption, last CD4 cell counts before TI, and prior ART regimen before TI (both based on the last available record as of 12 months before the date of interruption) were also included. The last ART regimen was categorized into the following four groups: TDF + NRTIs + NNRTIs, AZT + NRTIs + NNRTIs, lopinavir/ritonavir (LVP/r) + NRTIs, and others.

### 2.5. Statistical analysis

Demographic and HIV/ART-related characteristics at ART initiation were summarized with numbers and percentages [No. (%)] for categorical variables and medians and interquartile ranges (IQR) for continuous variables. The demographic and clinical characteristics of patients who discontinued and those who did not were compared using chi-square statistics. The incidence rate of TI was calculated as the number of events divided by the person-years of follow-up (PYFU) and was expressed as events per 100 PYFU.

Cox proportional hazards models were used to explore the association of TI with sociodemographic factors and HIV/ART-related characteristics. All variables were categorical and met the proportional hazards assumption. Variables with a *P*-value ≤ 0.05 in the univariate model were included in the multivariable analysis, and results were presented in forest plots. We used the Kaplan-Meier survival curve to illustrate the cumulative probability of TI 5 years after ART initiation, stratified by significant variables based on the above multivariable analysis.

For participants who had TI, Kaplan-Meier survival methods were used to estimate the probabilities of discontinuing during the 48 weeks since the date of TI. We used logistic regression to examine factors associated with ART resumption within 16 weeks. Missing values for the last CD4 cell counts were included in the analyses as a separate category (category “missing”). To assess the presence of multicollinearity among the variables, we ran the variance inflation factor (VIF) test, and all VIFs were low (i.e., <1.5), ensuring that multicollinearity was not a problem in the regression models. Variables with a *P*-value ≤ 0.05 in the univariate model were included in a multivariable analysis. The univariate and multivariable analysis results were presented graphically using forest plots.

All statistical analyses were performed with SAS statistical software (version 9.4) and R (v4.2.0). Two-sided *P*-values ≤ 0.05 were regarded as statistically significant.

## 3. Results

### 3.1. Study population

Of the adult PLHIV who had initiated ART in Jinan as of December 2020, 2506 were eligible for this study (Supplementary Figure S1), and 95% were male. At ART initiation, the median age of participants was 31 (IQR: 26–40) years, and over half of the participants (56%) were unmarried. More than two-thirds of subjects (77%) had high school education or higher, and most (91%) were at WHO clinical stage 1. The median nadir CD4 cell count was 338 (IQR: 206–480) cells/μL, and about half of the participants (48%) had a CD4 cell count >350 cells/μL at baseline. About half of the participants (45%) began ART within 30 days after HIV diagnosis. Nearly two-thirds of participants [1,619 (65%)] were prescribed a combination of TDF + NRTIs + NNRTIs.

Table 1 summarizes the demographic and clinical characteristics of PLHIV at ART initiation. Compared to non-interrupters, treatment interrupters were more likely to be female, with lower education levels, and unemployed (all *P*-values < 0.05). Baseline clinical factors that were more common among interrupters were heterosexual transmission, higher WHO clinical stage (2 and above), receiving ART care at the CDC, duration from HIV diagnosis to ART initiation > 90 days, and using “AZT+NRTIs+NNRTIs” and “E/C/F/TAF” as initial ART regimen (all *P*-values < 0.05).

**Table 1:** Sociodemographic and baseline clinical characteristics of HIV-positive adults in ART care.

**Characteristics**	**Total (*N* = 2,506)**	**Treatment interruption, No. (%)**		**χ^2^**	***P*** **-value**
		**No (*****n*** = **2,194)**	**Yes (*****n*** = **312)**		
Gender				5.706	0.017
Male	2,382 (95.05)	2,094 (95.44)	288 (92.31)		
Female	124 (4.95)	100 (4.56)	24 (7.69)		
Age group (years)				3.488	0.175
18–30	1,136 (45.33)	1,008 (45.94)	128 (41.03)		
31–40	778 (31.05)	679 (30.95)	99 (31.73)		
>40	592 (23.62)	507 (23.11)	85 (27.24)		
Age (years), median (IQR)	31 (26,40)	31 (26,40)	32 (27,42)		
Marriage status				6.515	0.011
Unmarried	1,413 (56.38)	1,258 (57.34)	155 (49.68)		
Married/ever married	1,093 (43.62)	936 (42.66)	157 (50.32)		
Education level^a^				12.664	< 0.001
Senior school and below	586 (23.45)	489 (22.32)	97 (31.49)		
High school and above	1,913 (76.55)	1,702 (77.68)	211 (68.51)		
Employment^b^				12.629	< 0.001
Employed	1,618 (64.64)	1,445 (65.92)	173 (55.63)		
Unemployed	885 (35.36)	747 (34.08)	138 (44.37)		
Transmission route				8.525	0.014
Heterosexual	359 (14.33)	298 (13.58)	61 (19.55)		
Homosexual	2,109 (84.16)	1,864 (84.96)	245 (78.53)		
Others	38 (1.52)	32 (1.46)	6 (1.92)		
WHO stage				6.09	0.014
I	2,272 (90.66)	2,001 (91.20)	271 (86.86)		
II or above	234 (9.34)	193 (8.80)	41 (13.14)		
Treatment facility				14.381	< 0.001
Hospital	2,158 (86.11)	1,911 (87.10)	247 (79.17)		
CDC	348 (13.89)	283 (12.90)	65 (20.83)		
Time from HIV diagnosis to ART initiation (days)				21.376	< 0.001
≤ 30	1,132 (45.17)	1,007 (45.90)	125 (40.06)		
31–90	591 (23.58)	536 (24.43)	55 (17.63)		
>90	783 (31.25)	651 (29.67)	132 (42.31)		
CD4 count category, cells/μL^c^				3.979	0.264
≤ 200	597 (23.87)	522 (23.84)	75 (24.12)		
201–350	716 (28.63)	614 (28.04)	102 (32.80)		
351–500	626 (25.03)	552 (25.21)	74 (23.79)		
>500	562 (22.47)	502 (22.92)	60 (19.29)		
CD4 count, cells/μL, median (IQR)	338 (206,480)	340 (208,482)	206 (320,466)		
ART regimen at baseline				31.747	< 0.001
TDF + NRTIs + NNRTIs	1,619 (64.60)	1,453 (66.23)	166 (53.21)		
AZT + NRTIs + NNRTIs	707 (28.21)	604 (27.53)	103 (33.01)		
E/C/F/TAF	94 (3.75)	71 (3.24)	23 (7.37)		
Others	86 (3.43)	66 (3.01)	20 (6.41)		

a 7 missing values.

b 3 missing values.

c 5 missing values.

HIV, human immunodeficiency virus; CDC, Centers for Disease Control and Prevention; ART, antiretroviral therapy; TDF, tenofovir; AZT, zidovudine; NRTIs, nucleoside reverse transcriptase inhibitors; NNRTIs, non-nucleoside reverse transcriptase inhibitors; E/C/F/TAF, Elvitegravir/cobicistat/Emtricitabine/Tenofovir alafenamide fumarate.

### 3.2. Time to first TI and associated factors

Over a median follow-up time of 3.4 (IQR: 1.7–5.3) years from baseline, 312 PLHIV (12.5%) had treatment interruption, corresponding to an incidence rate of TI of 3.2 (95% CI: 2.8–3.6) per 100 PYFU. Of all first TI, 143 (46%) occurred within the first 6 months after ART initiation; 38 (12%) occurred between 7 and 12 months; 61 (20%) occurred between 1 and 2 years, and 70 (22%) occurred between 3 and 5 years after ART initiation. In terms of duration of TI, 171 (55%) individuals were interrupted for < 6 months, 37(12%) were interrupted between 6 months and a year, 41 (13%) individuals were interrupted for 1–2 years, and 63 individuals (20%) had TI longer than 2 years.

**Figure 1:**
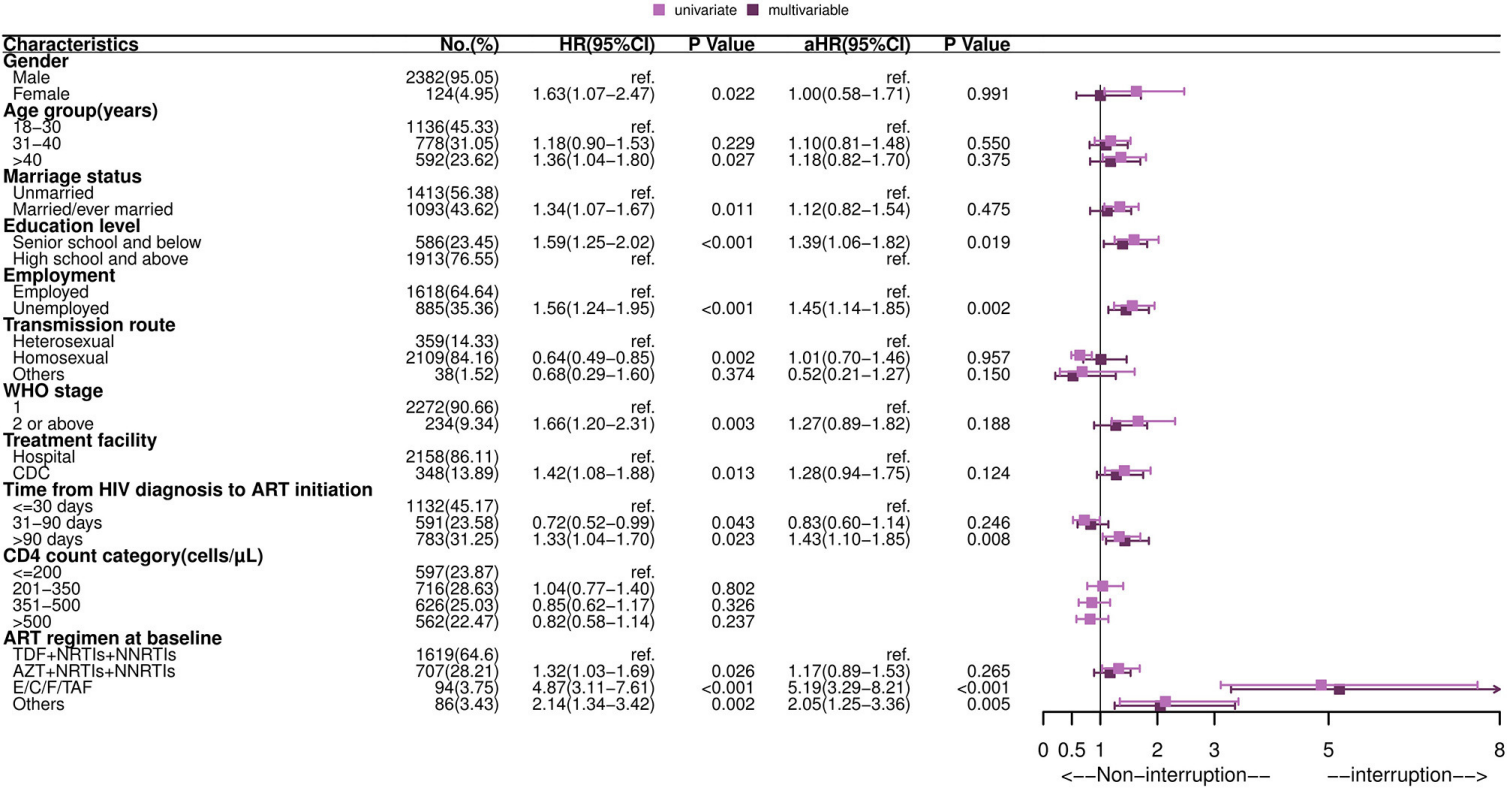
Forest plot of univariate and multivariable Cox regression of the first treatment interruption among HIV-infected adults. HR, hazard ratio; aHR, adjusted hazard ratio; CI, confidence interval; ART, antiretroviral therapy; TDF, tenofovir; AZT, zidovudine; NRTIs, nucleoside reverse transcriptase inhibitors; NNRTIs, non-nucleoside reverse transcriptase inhibitors; E/C/F/TAF, Elvitegravir/cobicistat/Emtricitabine/Tenofovir alafenamide fumarate. Multivariable analysis: factors with *P*-values ≤ 0.05 in univariate analysis were included in multivariable Cox regression analysis, including gender, age group, marriage status, education level, employment, transmission route, WHO stage, treatment facility, time from HIV diagnosis to ART initiation and drug regimen at baseline.

**Figure 2:**
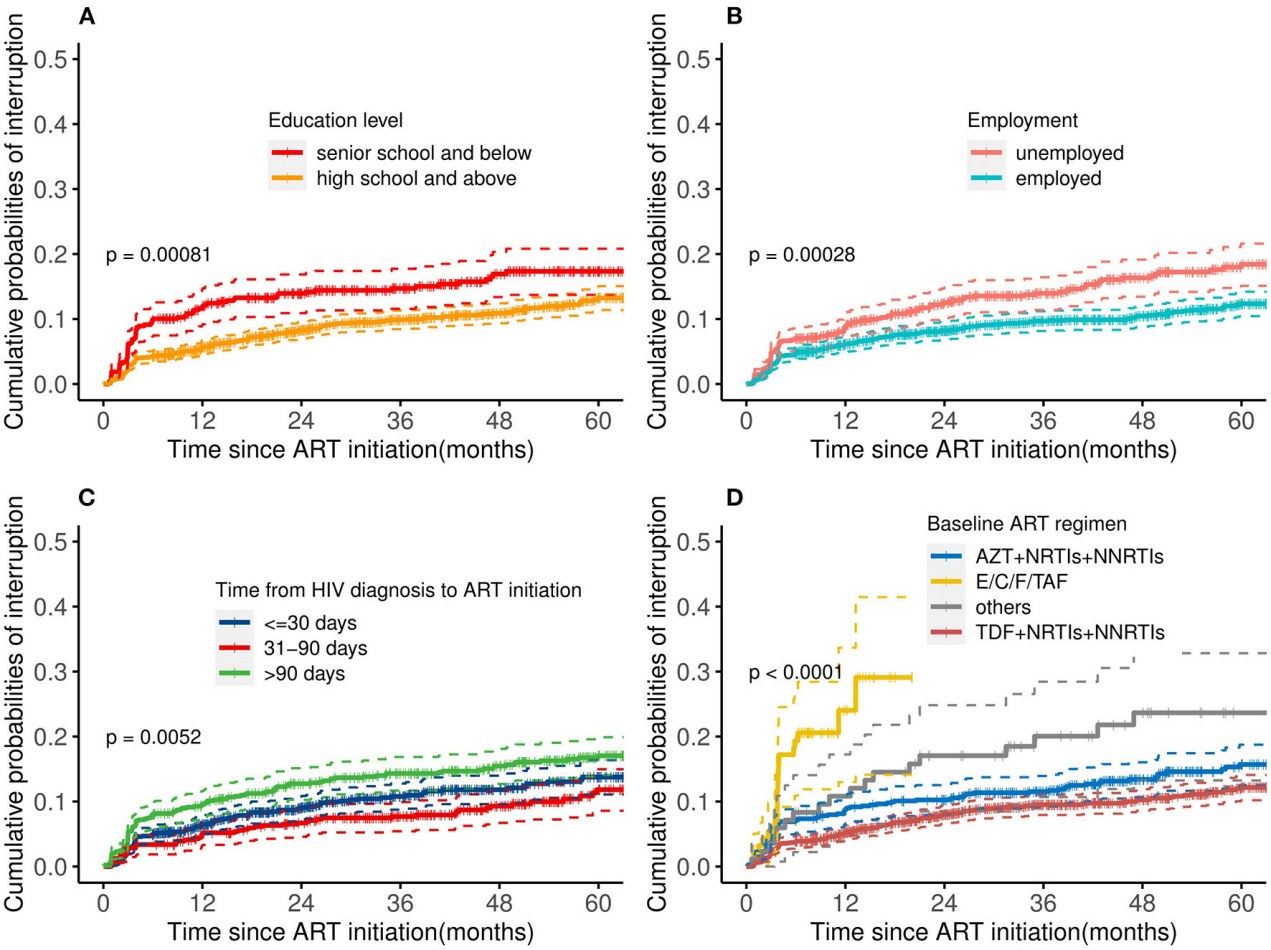
Cumulative probability of treatment interruption stratified by risk factors: **(A)** education level, **(B)** employment, **(C)** time between HIV diagnosis and ART initiation, and **(D)** baseline ART regimen. ART, antiretroviral therapy; AZT, zidovudine; TDF, tenofovir; NRTIs, nucleoside reverse transcriptase inhibitors; NNRTIs, non-nucleoside reverse transcriptase inhibitors; E/C/F/TAF, Elvitegravir/cobicistat/Emtricitabine/Tenofovir alafenamide fumarate. Log-rank tests were used to measure the statistical difference.

As shown in Figure 1, predictors of TI included a lower education level [adjusted hazard ratio (aHR): 1.39, 95% CI: 1.06–1.82], unemployed (aHR: 1.45, 95% CI: 1.14–1.85), time from HIV diagnosis to ART initiation more than 90 days (aHR: 1.43, 95%CI: 1.10–1.85) vs. <30 days, and use of an E/C/F/TAF drug (aHR: 5.19, 95% CI: 3.29–8.21) vs. “AZT+NRTIs+NNRTIs” regimen at ART initiation. Kaplan-Meier curves showed that the cumulative probability of discontinuation was greater among PLHIV with a low education level, who were unemployed, who had delayed initiation of ART (>90 days), and who used the E/C/F/TAF drug (all *P*-values < 0.05, see Figure 2). Particularly, the cumulative probability of interruption in the first year for participants who received E/C/F/TAF drugs was >20%.

### 3.3. ART resumption and barriers

**Figure 3:**
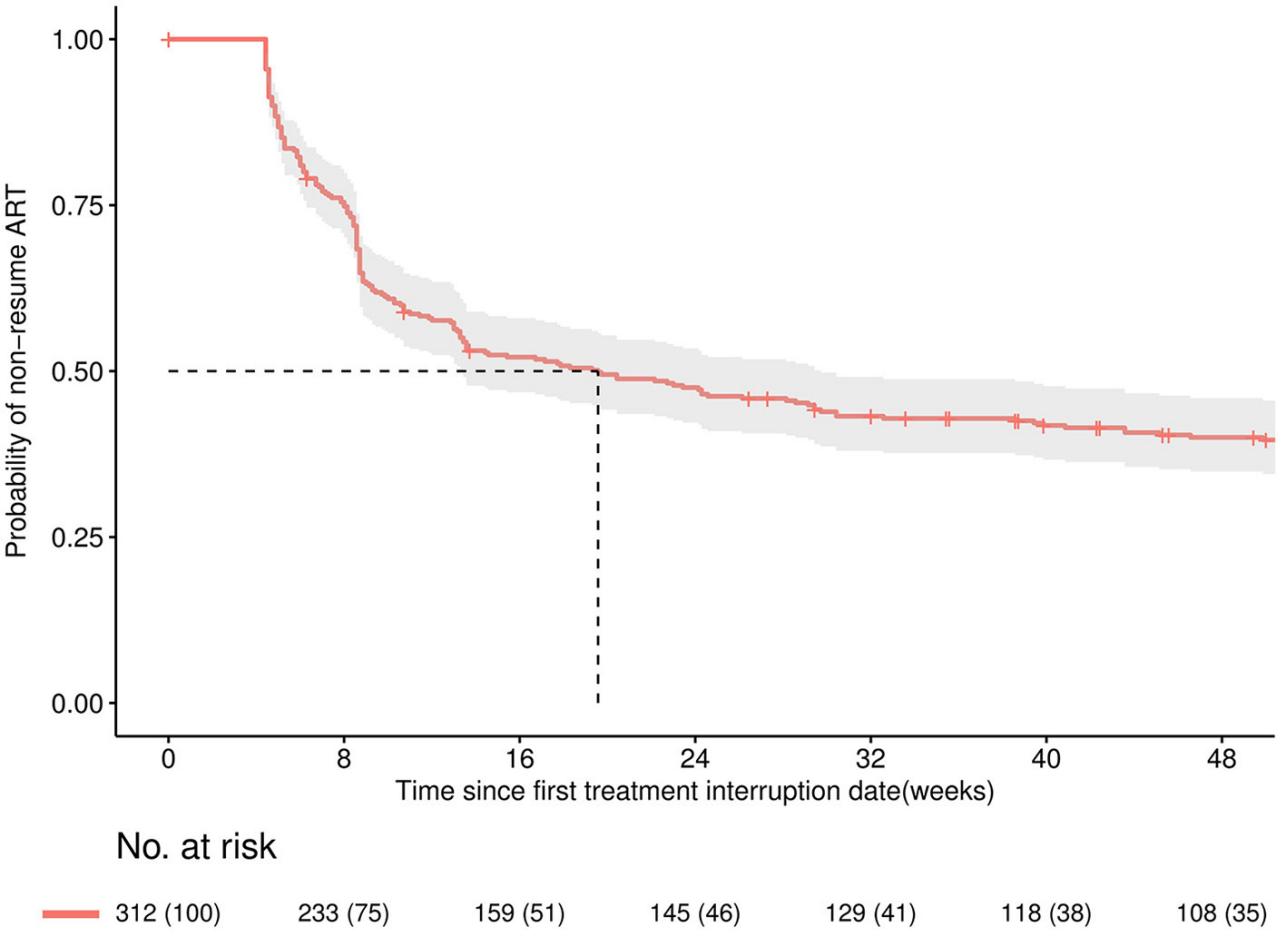
Kaplan-Meier curve for the probability of remaining discontinued within 48 weeks since the date of first treatment interruption. The solid line is the Kaplan-Meier estimated overall curve, shaded area is the 95% confidence band.

**Figure 4:**
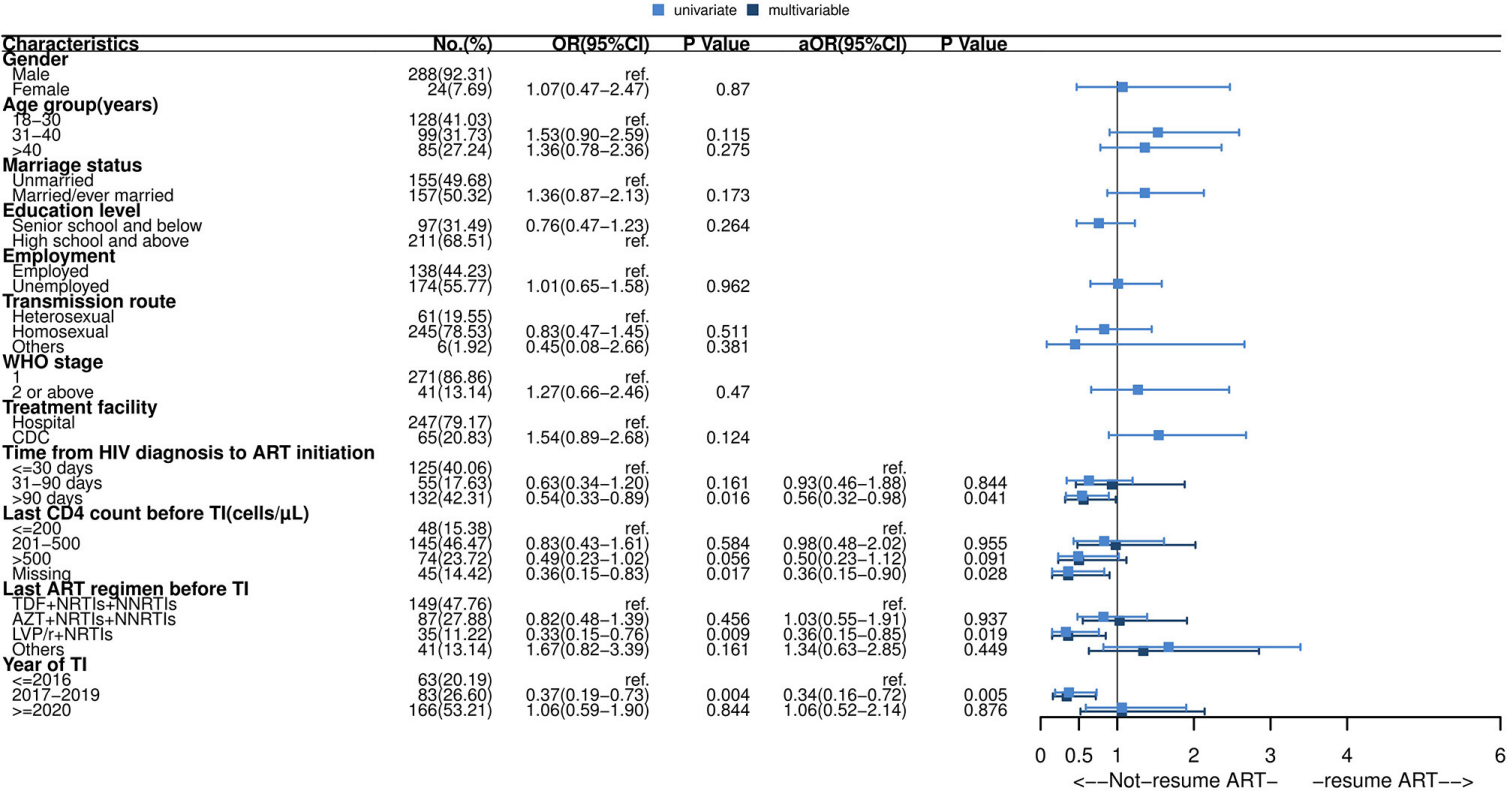
Forest plot of univariate and multivariable logistic regression of ART resumption after short-term interruption. OR, odds ratio; aOR, adjusted odds ratio; CI, confidence interval; ART, antiretroviral therapy; TDF, tenofovir; AZT, zidovudine; NRTIs, nucleoside reverse transcriptase inhibitors; NNRTIs, non-nucleoside reverse transcriptase inhibitors; LVP/r, lopinavir/ritonavir. Multivariable analysis: factors with *P*-values ≤ 0.05 in univariate analysis were included in multivariable logistic regression analysis, including time from HIV diagnosis to ART initiation, last CD4 count before interruption, last ART regimen before interruption and year of interruption.

Of the 312 individuals with TI, 151 (48.3%) restarted ART within 16 weeks. Among 151 patients who re-initiated ART, 135 (89.4%) kept the same previous regimen, and 16 (10.5%) changed their regimen. Details are shown in the Supplementary material. As presented in Figure 3, the median time to ART resumption was approximately 20 weeks. Resuming ART within 16 weeks was less likely to occur among patients with delayed ART initiation (>90 days) (aOR: 0.56, 95% CI: 0.32–0.98), missing the last CD4 test before TI (aOR: 0.36, 95% CI: 0.15–0.90), using an LVP/r + NRTIs regimen before TI (aOR: 0.36, 95% CI: 0.15–0.85) or interruption occurred during 2017–2019 (aOR: 0.34, 95% CI: 0.16–0.72) (see Figure 4).

## 4. Discussion

This study provided valuable real-world insights into the treatment interruption and resumption of ART in an HIV-positive adult cohort in China. The incidence rate of TI was 3.2 per 100 person-years, slightly higher than that of European countries (17, 18), similar to the Australian cohort (3.5 per 100 person-years) (33), lower than that of South African adults (12.8 per 100 person-years) (34). Factors associated with TI included unemployment, a lower education level, delayed initiation of ART (more than 90 days), and receiving the E/C/F/TAF drug at ART initiation, suggesting that TI more often occurred among PLHIV with a lower socioeconomic status. For PLHIV with TI, those who missed the last CD4 test before TI received the “LPV/r+NRTIs” regimen before TI, discontinued during 2017–2019, were less likely to return to care within 16 weeks, emphasizing that more focus should be on patients with the above risk characteristics to ensure that patients resume and experience the long-term benefits of ART.

Most discontinuations occurred during the first year after ART initiation, especially during the first 6 months, in line with prior study (35). For factors associated with TI, unemployment and a lower education level were positively correlated with interruption, as found in other studies (16, 19). It is conceivable that PLHIV who lack stable employment, also meaning lack of income, accompanied by irregular lifestyles during the unemployed period, may be unable to prioritize treatment adherence, resulting in ART interruptions. In univariate analysis, we found that participants who married/ever married were more likely to discontinue treatment due to the desire to conceal the infection status from the cohabitant. Therefore, we speculated that patients who were poor or had low education levels were more likely to live with others and may be discontinued because they wanted to hide their HIV status from their cohabitant. However, the association between age and gender with treatment interruption is inconsistent across studies (20–22). Age and gender were not statistically significantly associated with discontinuation when adjusted for other variables in our study. The discordant results may be partially attributable to a different composition of ages and gender among the study populations. In Jinan, a higher proportion of PLHIV at ART care were young and middle-aged males, which may be why age and gender were not significantly associated with TI.

The results of this study also suggest that delayed initiation of ART is a potential marker of treatment interruption. The reasons for these delays are complex and involve a combination of individual characteristics, psychological, social, and structural factors, and poor healthcare infrastructure in some settings (36). The interval time to start ART has been found to predict adherence in previous studies (37, 38), suggesting that health providers must pay more attention to adherence education and timely follow-up for PLHIV who delay ART initiation. It is recommended that clinicians should inquire about the reasons for the patient's delay and provide targeted psychological or physical support.

Free ART regimen offered in China combines the following three drugs: NRTIs, NNRTIs, and PIs, but single-tablet regimens (STRs) requires self-payment. In line with prior studies, PLHIV receiving AZT + NRTIs + NNRTIs regimens were more likely to discontinue due to side effects than TDF + NRTIs + NNRTIs regimens (23), but there were no significant differences between TI and AZT + NRTIs + NNRTIs regimens in this study after adjusted for other covariates. Unexpectedly, using the E\C\F\TAF (one common kind of STRs) drug was strongly associated with discontinuation. Although the inclusion of E\C\F\TAF in the Chinese national basic medical insurance program since December 2019 has significantly reduced its price, the out-of-pocket part is still a considerable cost for patients (~$193 per month) (39). Consistent with previous studies, discontinuation may be due to an increase in the patient's out-of-pocket part (24). As more STRs are included in basic medical insurance, more patients will likely choose to take these drugs due to fewer side effects and lower pill burden, but the accompanying higher out-of-pocket costs may cause poor adherence, which will remain an issue in the future. As guidelines for HIV care recommend, a routine assessment at entry into HIV care of new patients is paramount (40).

Almost half of the interrupters returned to care within 16 weeks, while the probability of resuming ART substantially reduced in the subsequent 32 weeks, suggesting clinicians should contact the patient who discontinued therapy as soon as possible to resume timely. Since China announced providing ART for PLHIV regardless of CD4 count in June 2016, the number of PLHIV enrolled in ART care has increased rapidly. Patients who discontinued treatment during 2017–2019 were less likely to resume therapy within 16 weeks, likely due to initial transition turmoil in the face of limited staff and a sudden increase in patients, providers neglecting to contact and remind patients who missed appointments.

Those who missed the last CD4 testing before the interruption had a significantly lower possibility of returning to care. This finding suggests that prompt and intensive intervention for returning to care is required in the case of discontinuation among patients who missed CD4 testing. Besides, patients who received “LPV/r+NRTIs” before TI were less likely to resume therapy; this may be due to some unbearable side effects, such as gastrointestinal side effects and fat redistribution among the LPV/r users, than the other medication (35).

There are some limitations to this study. First, we relied on prescription fill records in NFATP to determine interruption, which might not reflect patients' true discontinuation behavior and lead to misclassification. However, studies have suggested that prescription fill records provide valid estimates of TI (41). Compared with patient recall and/or physician documentation, a determination based on pharmacy records is more convenient and reduces recall bias. Besides, since the NFATP provides free consultation and subsidy policies for patients, most patients would not purchase drugs from other sources. Second, due to data limitations, we could not analyze the impact of the COVID-19 epidemic on drug discontinuation. ART treatment units adapted care models quickly to allow patients to engage in care without requiring in-person visits (42). Third, our analysis of factors associated with TI and ART resumption was restricted to information available in the data from NFATP. Other factors such as side effects, level of adherence information, resistance tests information, HIV status disclosure and HIV infection status of a partner, and aspects related to the external environment (23) may also play a key role. Further investigation is needed to determine.

## 5. Conclusion

Treatment interruption among HIV-positive adults remains relatively prevalent in the “treat all” era, often occurring in the first year of ART. Specific strategies may be warranted to decrease the occurrence of TI, particularly among PLHIV unemployed, with lower education levels, delayed initiation of ART, and use of E\C\F\TAF at baseline. Assessing the socioeconomic status and risk factors identified in this study at entry into HIV care among new patients should help address this issue. On the other hand, while it is reassuring to see that about half of the observed TI returned to care within 16 weeks after TI, patients who missed the last CD4 test before TI, received an “LPV/r+NRTIs” regimen before discontinuation were less likely to resume ART after a short-term interruption. Further targeted efforts are required to maximize the resumption of ART as soon as possible.

## Data availability statement

The data analyzed in this study is subject to the following licenses/restrictions: confidentiality and ethic reasons. Requests to access these datasets should be directed to WM, weima@sdu.edu.cn.

## Ethics statement

The studies involving human participants were reviewed and approved by Shandong University Ethics Review Committee of Public Health (IRB No: 20220806). Written informed consent for participation was not required for this study in accordance with the national legislation and the institutional requirements.

## Author contributions

JM developed the research concept, performed statistical analyses, and drafted the manuscript. XL and YJ provided oversight to data collection and helped with data management. KJ and YW contributed to data interpretation and discussion of the manuscript. WM and LG helped with the research concept and editing of the writing. WM, LG, and XL supervised the study. WM obtained funding and revised the manuscript. All authors contributed to the article and approved the submitted version.
