# Supplementary material for: Antiretroviral treatment interruption and resumption within 16 weeks among HIV-positive adults in Jinan, China: a retrospective cohort study
Source: Front Public Health. 2023 May 9;11:1137132. doi: 10.3389/fpubh.2023.1137132 (PMC10203161; doi:10.3389/fpubh.2023.1137132)
Supplement: Supplementary file 1 [file Data_Sheet_1.docx]

1. **Flow chart of study subject**

Participants included (N=2506)

阿甘

Adult HIV patients who started ART in Jinan from 2004 to 2020, without transferring out record (N=2515)

First treatment interruption

Followed up to Jun 2021

YES(N=312)

NO(N=2194)

COX regression

Followed up to Dec 2021

Resumption after 16 weeks interruption

NO(N=161)

YES(N=151)

Logistic regression

阿甘

Excluded:

Missing follow-up records (9)

Figure S1 Flow chart of study subject

1. **Results of univariate and multivariable Cox regression of treatment interruption among PLWHs stratified by gender.**

Table S1 Univariate and multivariable Cox regression of treatment interruption among PLWHs stratified by gender

| Characteristics | Male | | | | Female | | | |
| --- | --- | --- | --- | --- | --- | --- | --- | --- |
|  | Univariate | | Multivariable | | Univariate | | Multivariable | |
|  | HR(95%CI) | P | HR(95%CI) | P | HR(95%CI) | P | HR(95%CI) | P |
| Age group(years) |  |  |  |  |  |  |  |  |
| 18-30 | ref. |  | ref. |  | ref. |  |  |  |
| 31-40 | 1.19(0.91-1.57) | 0.201 | 1.09(0.80-1.50) | 0.577 | 0.59(0.21-1.69) | 0.325 |  |  |
| >40 | 1.36(1.02-1.81) | 0.038 | 1.14(0.77-1.68) | 0.520 | 0.72(0.28-1.91) | 0.513 |  |  |
| Marriage status |  |  |  |  |  |  |  |  |
| Unmarried | ref. |  | ref. |  | ref. |  |  |  |
| Married/ever married | 1.32(1.04-1.66) | 0.021 | 1.17(0.85-1.62) | 0.333 | 0.63(0.22-1.84) | 0.398 |  |  |
| Education level ^a^ |  |  |  |  |  |  |  |  |
| Senior school and below | 1.55(1.19-2.00) | 0.001 | 1.43(1.08-1.898) | 0.013 | 1.42(0.58-3.44) | 0.444 |  |  |
| High school and above | ref. |  | ref. |  | ref. |  |  |  |
| Employment ^b^ |  |  |  |  |  |  |  |  |
| Employed | ref. |  | ref. |  | ref. |  |  |  |
| Unemployed | 1.48(1.17-1.88) | 0.001 | 1.46(1.14-1.868) | 0.003 | 2.04(0.70-5.97) | 0.193 |  |  |
| Transmission route |  |  |  |  |  |  |  |  |
| Heterosexual | ref. |  | ref. |  | ref. |  |  |  |
| Homosexual | 0.68(0.48-0.95) | 0.024 | 1.00(0.69-1.44) | 0.985 | 0 | 0.992 |  |  |
| Others | 0.50(0.17-1.45) | 0.204 | 0.35(0.12-1.05) | 0.062 | 1.57(0.37-6.68) | 0.543 |  |  |
| WHO stage |  |  |  |  |  |  |  |  |
| Ⅰ | ref. |  | ref. |  | ref. |  | ref. |  |
| Ⅱ or above | 1.50(1.05-2.13) | 0.027 | 1.12(0.76-1.64) | 0.574 | 4.44(1.76-11.25) | 0.002 | 3.39(1.24-9.29) | 0.017 |
| Table S2 (Continued) |  |  |  |  |  |  |  |  |
| Characteristics | Male | | | | Female | | | |
|  | Univariate | | Multivariable | | Univariate | | Multivariable | |
|  | HR(95%CI) | P | HR(95%CI) | P | HR(95%CI) | P | HR(95%CI) | P |
| Treatment facility |  |  |  |  |  |  |  |  |
| Hospital | ref. |  | ref. |  | ref. |  |  |  |
| CDC | 1.49(1.12-1.99) | 0.006 | 1.39(1.01-1.92) | 0.042 | 0.69(0.20-2.34) | 0.550 |  |  |
| Time from HIV diagnosis to ART initiation (days) |  |  |  |  |  |  |  |  |
| ≤30 | ref. |  | ref. |  | ref. |  | ref. |  |
| 31-90 | 0.71(0.51-0.98) | 0.038 | 0.81(0.58-1.13) | 0.213 | 0.96(0.19-4.96) | 0.963 | 1.12(0.22-5.84) | 0.894 |
| >90 | 1.24(0.96-1.61) | 0.102 | 1.40(1.07-1.84) | 0.015 | 2.68(0.99-7.28) | 0.053 | 1.81(0.60-5.44) | 0.292 |
| CD4 count category(cells/µL) ^c^ |  |  |  |  |  |  |  |  |
| ≤200 | ref. |  |  |  | ref. |  |  |  |
| 201-350 | 1.07(0.78-1.46) | 0.695 |  |  | 0.84(0.30-2.32) | 0.734 |  |  |
| 351-500 | 0.88(0.63-1.22) | 0.437 |  |  | 0.70(0.21-2.40) | 0.571 |  |  |
| >500 | 0.82(0.57-1.16) | 0.260 |  |  | 0.91(0.29-2.87) | 0.874 |  |  |
| ART regimen at baseline |  |  |  |  |  |  |  |  |
| TDF+NRTIs+NNRTIs | ref. |  | ref. |  | ref. |  | ref. |  |
| AZT+NRTIs+NNRTIs | 1.31(1.02-1.70) | 0.037 | 1.17(0.89-1.54) | 0.270 | 1.62(0.63-4.19) | 0.318 | 1.12(0.40-3.14) | 0.828 |
| E/C/F/TAF | 4.42(2.75-7.13) | <0.001 | 4.77(2.93-7.78) | <0.001 | 26.32(6.25-110.86) | <0.001 | 12.95(2.80-59.97) | 0.001 |
| Others | 2.04(1.23-3.39) | 0.006 | 2.22(1.32-3.75) | 0.003 | 2.33(0.65-8.37) | 0.195 | 2.02(0.52-7.81) | 0.309 |

^a^ 7 missing values.

^b^ 3 missing values.

^c^ 5 missing values.

Abbreviations: HR, hazard ratio; aHR, adjusted hazard ratio; CI, confidence interval; ART, antiretroviral therapy; TDF, tenofovir; AZT, zidovudine; NRTIs, nucleoside reverse transcriptase inhibitors; NNRTIs, nonnucleoside reverse transcriptase inhibitors; E/C/F/TAF, Elvitegravir/cobicistat/Emtricitabine/Tenofovir alafenamide fumarate.

1. **Description of ART regimen after resumption among patients interrupted treatment**

Among 151 patients who re-initiated ART, 135 (89.4%) kept the same previous regimen, and 16 (10.5%) changed their regimen. Of 135 participants who kept the same regimen after resumption of therapy, 71 (52.5%) received TDF+NRTIs+NNRTIs regimen, 31 (22.9%) received AZT+NRTIs+NNRTIs regimen, 19 (14%) received E/C/F/TAF, 9 (6.7%) used LPV/r+NRTIs regimen and others (5, 3.7%). Of 16 subjects who changed regimen after resumption of treatment, 5 (31.3%) patients changed from AZT+NRTIs+NNRTIs to TDF+NRTIs+NNRTIs , 2 (12.5%) patients changed from AZT+NRTIs+NNRTIs to AZT+NRTIs+NNRTIs, 3 (18.7%) patients changed from TDF+NRTIs+NNRTIs to LPV/r+NRTIs regimen, as shown in Table S2.

Table S2 The type and number of changed regimen among 16 patients who re-initiated ART.

| Regimen before interruption | Regimen after ART resumption | Number |
| --- | --- | --- |
| AZT+NRTIs+NNRTIs | TDF+NRTIs+NNRTIs | 5 |
|  | AZT+NRTIs+NNRTIs | 2 |
|  | LPV/r+NRTIs | 1 |
|  | Others | 1 |
| TDF+NRTIs+NNRTIs | LPV/r+NRTIs | 3 |
|  | E/C/F/TAF | 1 |
|  | TDF+NRTIs+NNRTIs | 1 |
|  | Others | 1 |
| E/C/F/TAF | TDF+NRTIs+NNRTIs | 1 |

1. **Percentage of virologic suppression after resumption among patients interrupted treatment**

Among 151 patients who re-initiated ART after an interruption, 122 (80.8%) reached virologic suppression (viral load ≤50 copies/ml ) during follow-up.
